# Bioinformatic analysis of CaBP/calneuron proteins reveals a family of highly conserved vertebrate Ca^2+^-binding proteins

**DOI:** 10.1186/1756-0500-3-118

**Published:** 2010-04-28

**Authors:** Hannah V McCue, Lee P Haynes, Robert D Burgoyne

**Affiliations:** 1 The Physiological Laboratory, School of Biomedical Sciences, University of Liverpool, Crown Street, Liverpool L69 3BX, UK

## Abstract

**Background:**

Ca^2+^-binding proteins are important for the transduction of Ca^2+ ^signals into physiological outcomes. As in calmodulin many of the Ca^2+^-binding proteins bind Ca^2+ ^through EF-hand motifs. Amongst the large number of EF-hand containing Ca^2+^-binding proteins are a subfamily expressed in neurons and retinal photoreceptors known as the CaBPs and the related calneuron proteins. These were suggested to be vertebrate specific but exactly which family members are expressed outside of mammalian species had not been examined.

**Findings:**

We have carried out a bioinformatic analysis to determine when members of this family arose and the conserved aspects of the protein family. Sequences of human members of the family obtained from GenBank were used in Blast searches to identify corresponding proteins encoded in other species using searches of non-redundant proteins, genome sequences and mRNA sequences. Sequences were aligned and compared using ClustalW. Some families of Ca^2+^-binding proteins are known to show a progressive expansion in gene number as organisms increase in complexity. In contrast, the results for CaBPs and calneurons showed that a full complement of CaBPs and calneurons are present in the teleost fish *Danio rerio *and possibly in cartilaginous fish. These findings suggest that the entire family of genes may have arisen at the same time during vertebrate evolution. Certain members of the family (for example the short form of CaBP1 and calneuron 1) are highly conserved suggesting essential functional roles.

**Conclusions:**

The findings support the designation of the calneurons as a distinct sub-family. While the gene number for CaBPs/calneurons does not increase, a distinctive evolutionary change in these proteins in vertebrates has been an increase in the number of splice variants present in mammals.

## Introduction

Many aspects of cellular function are regulated by changes in the concentration of intracellular free Ca^2+ ^([Ca^2+^]i) [1]. Increased [Ca^2+^]i leads to changes in the Ca^2+ ^loading of various Ca^2+^-binding proteins [2]. In the case of those proteins that act as Ca^2+ ^sensors, Ca^2+ ^binding results in a significant conformational change that can expose sites for the interaction of target proteins [3]. Regulation of the function of the target proteins results in a wide range of physiological changes. Ca^2+ ^signals in cells can vary in their amplitude, timing and spatial localisation [4,5] and this in part contributes to how changes in the concentration of a single ion can lead to a multitude of physiological outcomes. Signalling specificity is also generated by the existence of multiple Ca^2+ ^sensors that have different properties and specific target proteins [3,6].

The ubiquitous protein calmodulin is the best known Ca^2+^-sensor [7]. It binds Ca^2+ ^through its four EF hand domains first identified in the Ca^2+^-buffer protein parvalbumin [8]. There are large numbers of known EF-hand containing proteins [9] some of which form distinct families. Examples of these are the S100 proteins in vertebrates [10] and the calcineurin-like (CBL) proteins in plants [11,12]. In neurons, Ca^2+ ^has multiple functional effects on timescales ranging from 10s of microseconds to many minutes and so neuronally expressed Ca^2+ ^sensors have been of particular interest. Two such families that have become more widely studied in recent years are the neuronal calcium sensor (NCS) [6,13] and the CaBP [14-17] proteins. One aspect that could provide important functional clues is an understanding of how these families have appeared and expanded during the evolution of increasingly complex organism behaviour. In the case of NCS proteins, a single gene known as frequenin or NCS-1 is encoded in fungal genomes and there has been a progressive expansion of the family during evolution. Three NCS proteins (similar to NCS-1) are expressed in *C. elegans*, whereas *D. melanogaster *has four NCS proteins that include two frequenins, a neurocalcin and a single Kv channel-interacting protein (KChIP). Zebrafish (D. rerio) have two NCS-1 orthologues, 8 visinin-like proteins (VILIPs), a recoverin, 8 guanylyl cyclase-activating proteins (GCAPs) and 5 KChIPs. All mammals have a highly conserved set of 14 NCS genes that encode one NCS-1, 5 VILIPs, one recoverin, three GCAPs and four KChIPs. There are in addition, multiple isoforms of KChIPs expressed in mammalian neurons generated by alternative splicing [18]. Analogously, the plant CBL proteins have also shown an increase in gene number during evolution. There is one CBL orthologue in algae and the CBLs increase progressively in number through mosses and onto higher plants [12]. These findings suggest that expansion of families of Ca^2+^-sensors by gene duplication may contribute to increased signalling complexity in higher organisms. The neuronally-expressed calcium-binding proteins (CaBPs) were thought to be expressed only in vertebrates [14]. Two of the proteins named CaBP7 and 8 [15] were also discovered independently and named calneuron 2 and 1 respectively [19,20]. While these latter two proteins were suggested to be part of the CaBP family [15], others have suggested that the calneurons are a distinct subgroup of the CaBPs [19,21]. The latter interpretation is consistent with the analyses described in the current paper and, therefore, we will refer to them henceforth as calneurons 1 and 2.

We have carried out a bioinformatic analysis of the vertebrate CaBPs/calneurons to gain insight into the evolution of this protein family. In contrast to the NCS protein families, all of the CaBP/calneuron genes may have arisen together. They are all expressed in the teleost fish *D. rerio *and the family members are present in evolutionary later species with the short isoform CaBP1 and calneuron 1, in particular, showing high levels of sequence conservation across species. The major evolutionary change after the teleost separation from tetrapods appears to be an increase in the number of splice variants of CaBPs in mammals.

## Methods

The accession numbers used for bioinformatic analysis of the human CaBP/calneuron proteins retrieved from the GenBank database http://www.ncbi.nlm.nih.gov/sites/entrez were: caldendrin (NM_001033677), CaBP1L (NM_031205), CaBP1S (NM_004276), CaBP2S (NM_031204), CaBP2L (NM_016366), CaBP4 (NM_145200), CaBP5 (NM_019855), CaBP7 or calneuron 2 (NM_182527), CaBP8 or calneuron 1 (AY007302) calmodulin (AAA35635). These sequences were used in BLASTP searches [22] using BLAST 2.2.22 at NCBI http://blast.ncbi.nlm.nih.gov/Blast.cgi against the database of non-redundant protein sequences for each organism individually. Further searches for missing CaBP sequences and for identified domains were carried out using TBLASTN searches against the Reference genomic sequence and the Reference mRNA sequence databases. The accession numbers of sequences used for analysis of Zebrafish (*Danio rerio*) proteins were: calmodulin (NP_955864), CaBP1 (NP_001002414), CaBP2 (NP_001025439), CaBP4 (XP_001338361), CaBP5 (NP_956992), CaBP7 or calneuron 2(XP_683885), CaBP8 or calneuron 1(XP_690899). The additional duplicated proteins identified in zebrafish were CaBP1 (NP_001005962), CaBP2 (XP_688066), CaBP5 (NP_00108568), CaBP7 or calneuron 2(XP_001920190), CaBP8 or calneuron 1(XP_001343041). The positions of the EF-hand motifs were identified by comparison to the EF-hand consensus sequence [8] and alignment with calmodulin. Searches within the genomes of other species were carried out on 21^st ^January 2010 at NCBI. Blast search of genomic sequences of the elephant shark (*Callorhinchus milli*) were carried out at http://esharkgenome.imcb.a-star.edu.sg/.

Multiple sequence alignments were compiled using ClustalW (matrix: BLOSUM62 and default settings) and the BioEdit Sequence Alignment Editor version 6.0.5 (@ Tom Hall, Ibis Therapeutics, Carlsbad, California; http://www.mbio.ncsu.edu/BioEdit/bioedit.html). Tables of identity values were generated using the Sequence Identity Matrix option in BioEdit. The open reading frames from the mRNA transcript of human calneuron 1 (CaBP8) isoform 1 (NM_031468.3) and 2 (NM_001017440.2) were found using BioEdit and the sequences surrounding the start sites analysed for the presence of a Kozak consensus sequence [23].

For the creation of a phylogenetic tree of human and zebrafish calneurons and CaBPs, the variable portion of the N-termini amino acid sequences were removed from each protein and ClustalW was used to create an alignment. The C-termini of the alignment was adjusted to create a more realistic representation of homology in this region. A maximum likelihood tree was created using the Phylip suite of programs. Seqboot.exe was used to make 2000 bootstrapped data sets from the alignment. Proml.exe was then used to find the phylogeny estimate for each of these data sets using the maximum likelihood method for protein amino acid sequences. Calmodulin was selected as the out-group root at this stage. A consensus tree was then created from the outtree file from Proml.exe using consense.exe. The final consensus tree was drawn using TreeView.

## Results

### Human CaBP/calneuron proteins

**Figure 1:**
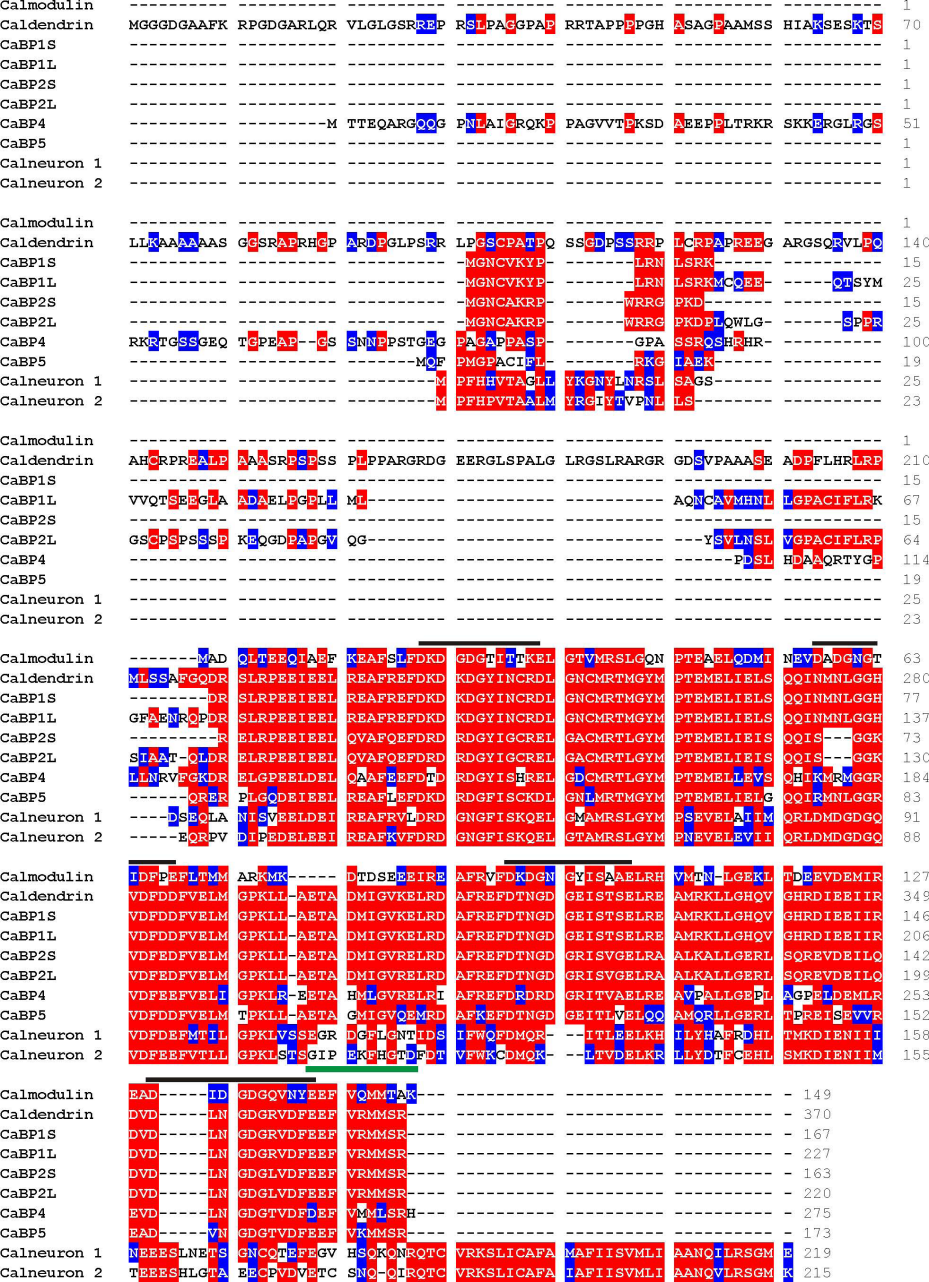
**Alignment of the sequences of human calmodulin, CaBPs and calneuron proteins**. Protein sequences were compiled and aligned using ClustalW. Residues that are identical in more than one protein are highlighted in red and residues with similar properties are highlighted in blue. The lines above the sequences indicate the position of the predicted 12 amino acid coordinating loop within the EF-hands The green bar beneath the sequence indicates the position of the linker region in the CaBPs that is extended in comparison to calmodulin.

The CaBP/calneuron family expressed in humans is derived from six genes. Two of these CaBP1 and CaBP2 give rise to three (caldendrin, CaBP1S, CaBP1L) or two (CaBP2S, CaBP2L) splice variants respectively (Figure 1). The human gene originally described to encode CaBP3 overlaps with the CaBP5 gene [14]. CaBP3 is found in human genomic sequences but in no other species and CaBP3 expression has not been detected [15]. CaBP3 is most likely, therefore, to be a pseudogene and is therefore not included in Figure 1. Note that despite the published numbering of CaBPs [15], CaBP6 has not been described.

Alternative transcripts of calneuron 1 were present in Genbank for *Homo sapiens *and a number of other species that could encode an N-terminally extended version of calneuron 1. The start codon in these transcripts was not surrounded by a Kozak consensus sequence suggesting that they may not be translated efficiently. In support of this suggestion, no evidence has been provided for the expression of a large form of calneuron 1 protein. We, therefore, did not include the long form in our analysis.

Figure 1 shows the multiple sequence alignment of the CaBP/calneuron protein sequences along with calmodulin. Residues which are identical or similar across the proteins are highlighted. The position of the four EF-hand loop domains in each protein is shown by the line above the sequences. The C-terminal portion of the proteins containing the EF-hand motifs is the most conserved region of these proteins with the N-terminal region being most divergent. Compared to calmodulin the CaBPs and calneurons have an extended linker region between EF hands 2 and 3 (underlined in green in Figure 1). There is a high level of identity in the CaBPs over this linker region but this region is not so similar in the calneurons. Some of the EF hands have undergone deletions or substitutions within their EF hand loop which mean they are likely unable to bind Ca^2+ ^or other cations at these positions. In caldendrin/CaBP1 several substitutions in the conventional EF hand motifs were predicted to prevent coordination of divalent cations by EF2 [14] and biochemical and structural analysis of CaBP1S confirmed that EF2 cannot bind Ca^2+ ^or Mg ^2+ ^[24,25]. The EF2 of CaBP2, 4 and 5 would also be predicted not to bind cations due to deletions or substitutions in the loop. In calneuron 1 and calneuron 2 there is a three amino acid deletion in EF3 and the binding loop of EF4 has both substitutions and insertions thus these proteins are predicted to only bind divalent cations using EF1 and EF2 [15,19].

Table 1 shows the percentage identity between each of the proteins in the multiple alignments of human proteins. The short forms of CaBP1, CaBP2, and CaBP5 exhibit the highest sequence similarity to calmodulin with around 36% identity. Overall Table 1 highlights that the CaBPs show only limited similarity with calmodulin. The source of this divergence lies within the N-terminal regions of the CaBPs and the members with longer N-termini have the least sequence identity to calmodulin. This table also shows that calneurons 1 and 2 have the lowest comparative identities within the CaBP family. Although they have 63% identity with each other, the maximum identity score which occurs between calneuron 2 and CaBP5 is only 24.7%. The identity scores between CaBPs 1-5 however suggest higher relatedness between these proteins. These values support the idea of two distinct sub-groups within the CaBP/calneuron family. Such a division is also supported by the presence of a 38 amino acid hydrophobic extension which occurs at the C-terminus of calneuron 1 and 2 that is missing from the other CaBP members and is important in membrane targeting [26]. In addition, a different pattern of active EF-hand motifs is seen in the calneurons with EF1 and EF2 being predicted as being high affinity Ca^2+ ^binding sites and EF3 and EF4 being unable to bind to Ca^2+^. In the rest of the CaBPs EF1, EF3 and EF4 are all predicted to be active in Ca^2+ ^binding.

**Table 1:** The degree of percentage sequence identity of residues across the various human calmodulin, CaBP and calneuron protein sequences that are shown in Figure 1.

	Caldendrin	CaBP1S	CaBP1L	CaBP2S	CaBP2L	CaBP4	CaBP5	Calneuron 1	Calneuron 2
Calmodulin	16.7	36.6	27.1	37.8	28.5	22.5	36.2	21.1	20.0
Caldendrin		42.7	45.9	32.4	35.6	32.3	30.8	13.3	12.4
CaBP1S			73.5	72.6	55.1	36.3	66.4	22.9	22.6
CaBP1L				53.5	60.5	33.1	50.2	18.3	18.3
CaBP2S					74.0	39.4	60.9	22.0	23.1
CaBP2L						37.3	47.1	17.8	18.9
CaBP4							34.1	16.0	16.0
CaBP5								23.6	24.2
Calneuron 1									63.0
Calneuron 2									

### Search for CaBPs in other species

In order to gain some insight into when the CaBPs first arose during evolution, database searches were carried out by performing BLAST searches of each of the human CaBP sequences in comparison to available sequence data. Specific, detailed searches were made in the NCBI databases for particular organisms. No proteins with significant homology greater than calmodulin were detected in *Drosophila *species or in *Caenorhabdities elegans *as representative invertebrate species. Searches of genomes of the early deuterostomes, the sea urchin (*Strongylocentrotus purpuratus)*, sea squirt (*Ciona intestinalis*) and amphioxus (*Branchiostoma floridae*) also did not identify any candidate CaBPs again with best matches to calmodulin or calmodulin-like proteins. Similarly no CaBP candidates were identified within lamprey or hagfish (cyclostomes) sequence data. A search of available sequence data for cartilaginous fish (Chondrichthyes) within NCBI did not detect the existence of proteins with clear homology to CaBPs apart from calmodulin but searches of the genomic data for the elephant shark (*Callorhinchus milli*) did identify 5 short sequences indicating the likely presence of at least three CaBPs related to CaBP1 and 5 and also the presence of both calneurons 1 and 2. The fragmentary nature of these data precluded further analysis. Full sequences of CaBPs and calneurons were identified in teleost genomes including puffer fish and zebrafish. We concentrated upon CaBP/calneurons identified in zebrafish but it is likely, however, that the CaBP/calneurons arose as a full family before the separation of cartilaginous and teleost fish.

### Zebrafish CaBP/calneuron proteins

**Figure 2:**
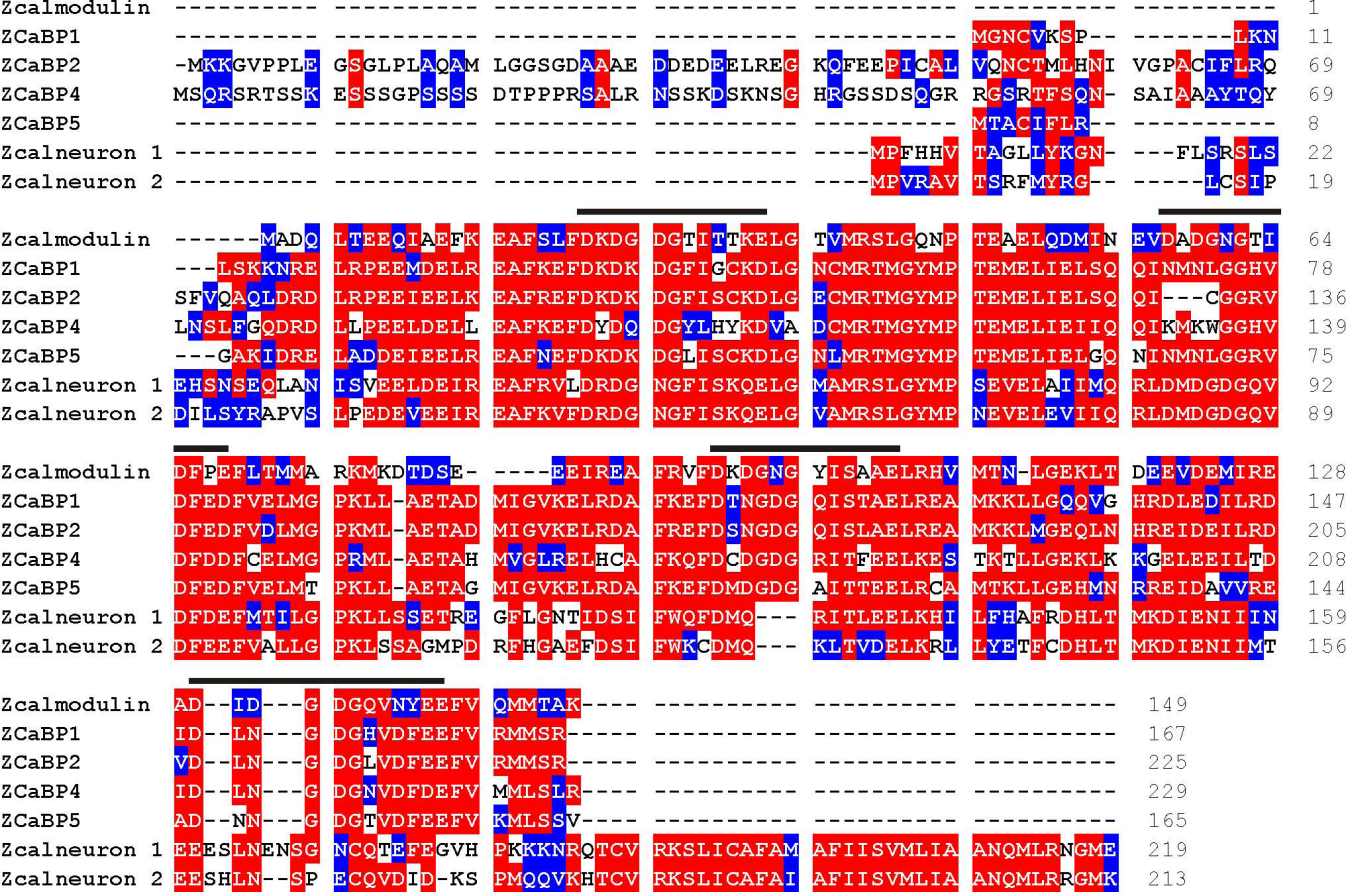
**Alignment of the sequences of zebrafish (*Danio rerio*) calmodulin, CaBPs and calneuron proteins**. Protein sequences were compiled and aligned using ClustalW. Residues that are identical in more than one protein are highlighted in red and residues with similar properties are highlighted in blue.

**Figure 3:**
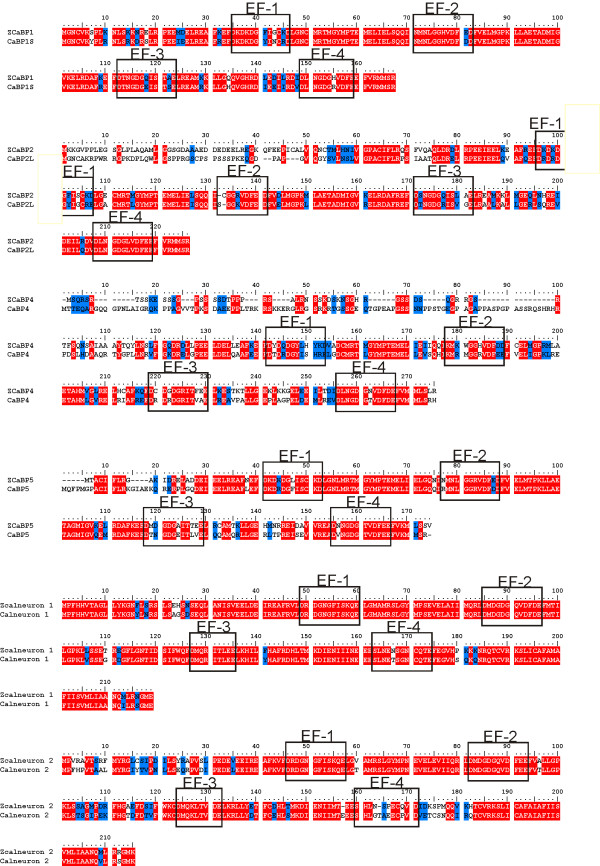
**Alignment of the sequences of the individual zebrafish and the corresponding human CaBPs and calneuron proteins**. Protein sequences were compiled and aligned using ClustalW. Residues that are identical in more than one protein are highlighted in red and residues with similar properties are highlighted in blue. The position of the predicted EF hands in each protein is indicated.

A complement of CaBP proteins could be detected in teleost fish and in fact the zebrafish genome encodes genes corresponding to all of the human CaBP/calneuron genes (Figure 2 and Tables 2, 3). The teleost genome underwent a whole genome duplication event [27] and we identified two separate genes for all of the CaBP/calneurons except CaBP4. To allow easier comparison with human sequences we selected zebrafish sequences with the highest similarity to their human counterparts. In Table 2 highlighted cells show the percentage identities between identified orthologues. Distinct long and short isoforms of CaBP1 and CaBP2 were not found in zebrafish but the highlighted cells show that the zebrafish CaBP1 is most like the human CaBP1 short form and the zebrafish CaBP2 is most like the human CaBP2 long form (Table 2). Over 60% identity between the zebrafish and human orthologues is seen for most family members but only 44.7% is seen in the case of CaBP4. This illustrates that the CaBPs are strongly evolutionarily conserved, particularly in the case of calneuron 1 which shows 93.1% sequence identity between zebrafish and human proteins. Other CaBPs show more divergence between zebrafish and humans (Table 2 and Figure 3).

**Table 2:** Comparison of the percentage sequence identity of zebrafish CaBP and calneuron proteins in relation to the human protein sequences.

		Zebrafish						
		**ZCalmodulin**	**ZCaBP1**	**ZCaBP2**	**ZCaBP4**	**ZCaBP5**	**ZCalneuron 1**	**ZCalneuron 2**
	Calmodulin	**100**	35.5	29.6	24.0	38.7	20.7	20.2
	Caldendrin	16.7	37.0	36.2	28.0	30.4	13.3	13.4
	CaBP1S	36.6	**86.8**	54.3	40.7	66.6	23.7	24.4
	CaBP1L	27.1	63.8	44.2	32.9	50.0	19.5	19.6
Human	CaBP2S	37.8	62.0	52.2	41.7	54.4	20.1	23.1
	CaBP2L	28.5	50.2	**60.6**	39.6	46.8	18.7	20.4
	CaBP4	22.9	35.4	38.1	**44.7**	34.5	17.0	16.8
	CaBP5	36.7	62.1	51.7	38.4	**73.5**	22.4	23.9
	Calneuron 1	20.2	22.4	17.7	18.4	21.0	**93.1**	59.3
	Calneuron 2	20.1	22.1	18.1	17.8	22.0	60.2	**77.2**

The closest matches between zebrafish and human proteins with the highest percentage similarity are shown in bold and are underlined.

**Table 3:** The degree of percentage sequence identity of residues across the various zebrafish calmodulin, CaBP and calneuron protein sequences that are shown in Figure 2.

	ZCaBP1	ZCaBP2	ZCaBP4	ZCaBP5	Zcalneuron 1	Zcalneuron 2
Zcalmodulin	34.9	29.1	23.4	37.9	20.7	21.1
ZCaBP1		55.7	44.1	68.4	23.8	24.8
ZCaBP2			43.9	49.3	19.7	20.2
ZCaBP4				38.8	20.8	18.3
ZCaBP5					22.5	24.4
Zcalneuron 1						59.3
Zcalneuron 2						

**Figure 4:**
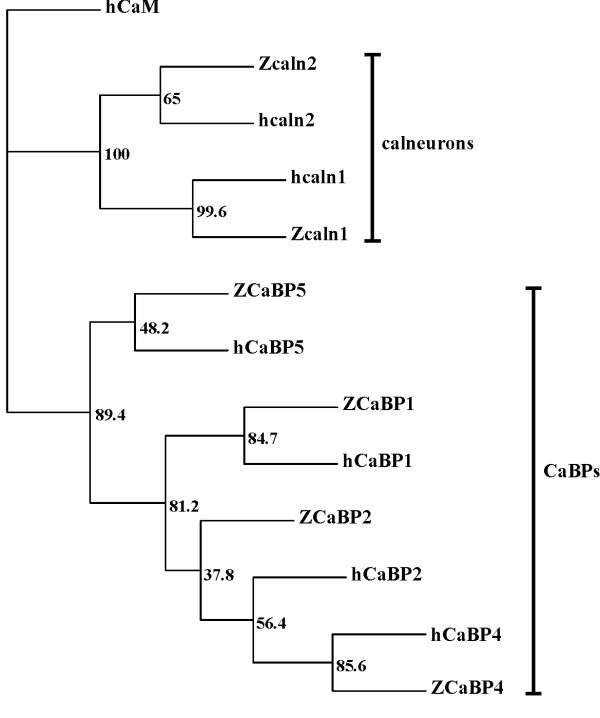
**Phylogenetic tree showing the relatedness of the human and zebrafish calmodulin, CaBPs and calneuron proteins**. A consensus maximum likelihood tree based on the alignment of the CaBP and calneuron proteins was generated and rooted on human calmodulin. The percentage of 2000 bootstrap replicates supporting the topology is given at each node.

A phylogenetic tree rooted on calmodulin was constructed based on the sequences of the human and zebrafish CaBPs and calneurons (Figure 4). For this analysis the N-terminal domains of each protein that in some cases vary between splice isoforms were excluded as they are highly variable and not well conserved suggesting that they are under less functional evolutionary pressure. Instead the core "calmodulin-like" domains were compared. The analysis divided the tree into two clades consisting of the CaBPs and the calneurons respectively. Amongst the CaBPs, CaBP2 and 4 form a distinct subgroup from CaBP1 and CaBP5.

As seen for the human CaBP proteins certain of the EF hands in the zebrafish proteins are predicted to be inactive in divalent cation binding and these were the same as those in the human proteins (Table 4). In addition to having EF hands with inactivating substitutions and deletions leading to impaired cation binding, certain CaBP EF hands are predicted to bind Mg^2+ ^rather than Ca^2+^. The nature of the residue at position 12 of the EF hand loop motif (the sequence in EF hand 1 of calmodulin for example being DKDGDGTITTKE) is crucial in that substitution of glutamate by aspartate has been shown to diminish binding of Ca^2+ ^in favour of Mg^2+^. CaBP1 is predicted to bind Mg^2+ ^rather than Ca^2+ ^at EF1 and this was confirmed in structural analyses that indicated that it is constitutively Mg^2+ ^bound whereas EF3 and EF4 can bind to both Ca^2+ ^and Mg^2+ ^[24,25]. Human CaBP5 also has an aspartate at position 12 in its predicted functional EF hand 1. We examined the degree of conservation of EF hand properties between zebrafish and human proteins. The same patterns of EF hand inactivation were observed for the proteins of both species (Table 4). Comparison of the amino acid at position 12 and the percentage identity of the EF hand loops indicated varying degrees of conservation. Overall the most conserved EF hand in the CaBPs was EF4. There were no differences at position 12, and thus in predicted Mg^2+ ^versus Ca^2+ ^binding, for the EF hands of CaBP1. In contrast, EF hands in CaBP2,4 and 5 were found to have the D substitution in zebrafish predicting Mg^2+ ^binding but E at this position in the human CaBP2 and 4 proteins suggesting an increase in the number of specific Ca^2+^-binding EF hands from zebrafish to man. It was noteworthy that the sequences of the predicted functional EF hands of the calneurons (EF1 and EF2) were 100% conserved from zebrafish to humans.

**Table 4:** Comparison of the EF hands in zebrafish and human CaBPs and calneurons.

	EF1			EF2			EF3			EF4		
	**Zebrafish**	**Human**	**Conserved**	**Zebrafish**	**Human**	**Conserved**	**Zebrafish**	**Human**	**Conserved**	**Zebrafish**	**Human**	**Conserved**
CaBP1	D	D	75%	D	D	92% (N)	E	E	83%	E	E	92%
CaBP2	D	E	50%	D	D	78% (N)	E	E	67%	E	E	100%
CaBP4	D	E	42%	D	E	58% (N)	E	E	67%	E	E	92%
CaBP5	D	D	58%	D	D	83% (N)	E	E	58%	E	E	92%
Calneuron 1	E	E	100%	E	E	100%	E	E	100% (N)	E	E	92% (N)
Calneuron 2	E	E	100%	E	E	100%	E	E	100% (N)	I	D	58% (N)

The table indicates whether the amino acid at position 12 of the EF-hand Ca^2+^-coordinating loop is an aspartic acid (D) or glutamate (E) and the percentage sequence identity between zebrafish and human EF-hand loop sequences. In the conserved column it is also indicated if the EF hand is predicted to be non-functional (N) and these are the same in the human and zebrafish proteins.

### CaBP/calneuron proteins of other species and evolutionary changes

The most conserved of the CaBP/calneuron proteins from zebrafish to human are in order: calneuron 1, CaBP1S and calneuron 2 (Table 2). The main differences between the two species were the lack of caldendrin, CaBP1L and CaBP2S splice variants in zebrafish and variability in the N-termini of CaBP2L and CaBP4. We compared the sequences of CaBPs from a wider range of species including zebrafinch (*Taeniopygia guttata*), chicken (*Gallus gallus*), frog (*Xenopus laevis*), opossum (*Monodelphis domestica)*, dog (*Canis lupus familiaris*), mouse (*Mus musculus*), rat (*Rattus norvegicus*), cow (*Bos taurus*), macaque (*Macaca mulatta*) and chimpanzee (*Pan troglodytes*) and observed a number of evolutionary changes in the protein family. We could not find evidence for either CaBP4 or CaBP5 in available amphibian or bird genomic or protein sequence databases but this may be a consequence of the incompleteness of the genome sequences for these species.

**Figure 5:**
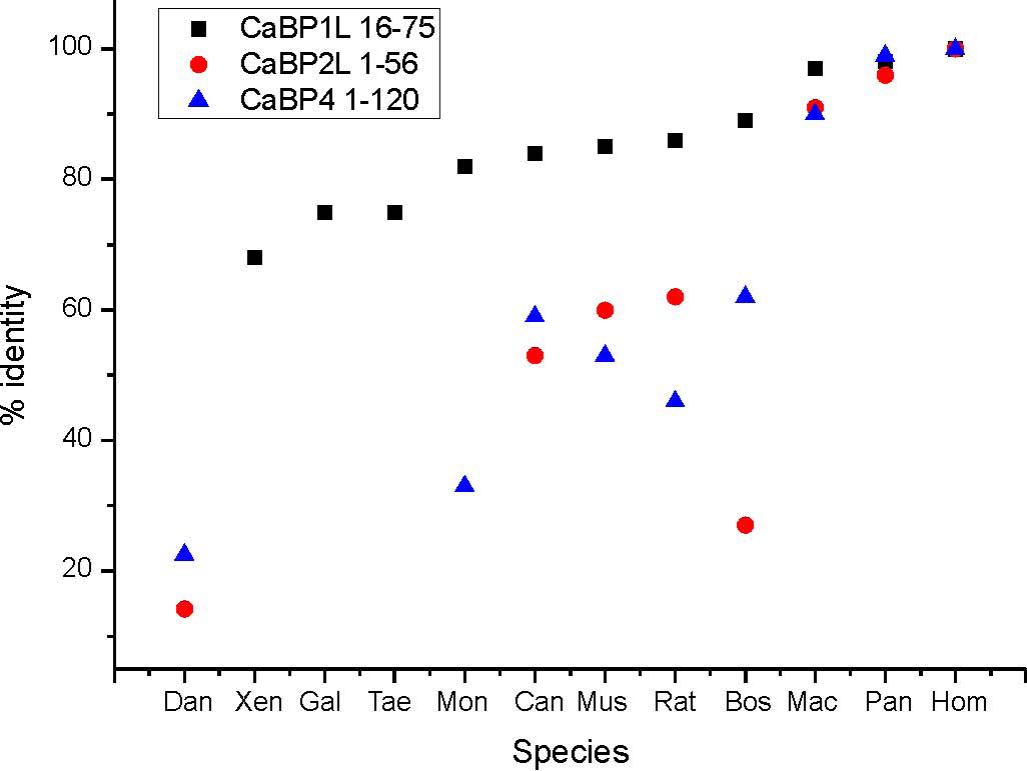
**Relative sequence identity of individual domains with the human sequence**. The percentage identity compared to human is shown for three indicated domains for a range of species. Where no symbol is present no orthologous protein or domain could be found from Blast searches. The following species are shown *Danio rerio *(Dan), *Xenopus laevis *(Xen), *Gallus gallus *(Gal), *Taeniopygia guttata *(Tae), *Monodelphis domestica *(Mon), *Canis lupus familiaris *(Can), *Mus musculus *(Mus), *Rattus norvegicus *(Rat), *Bos taurus *(Bos), *Macaca mulatta *(Mac) *Pan troglodytes *(Pan) and *Homo sapiens *(Hom).

The extended N-terminal domain of caldendrin is encoded by mammalian but not other genomes. The relative sequence identity of other relevant domains in the CaBPs is compared in Figure 5 and these show different patterns of evolutionary change. The domain that defines CaBP1L (CaBP1L16-75) which was not in the zebrafish was present from Xenopus onwards with relatively little subsequent change in sequence identity compared to human caldendrin. The variable N-terminal domain of CaBL2L (1-56) was not found in available amphibian, bird or marsupial genomic sequences. In mammalian species this domain showed a progressive increase in similarity to the human protein. The variable N-terminal region of CaBP4 (1-120) was present in marsupials and also showed a progressive increase in sequence identity with the human protein.

### Expression of target proteins for the CaBP/calneuron proteins

A number of target proteins are known for the CaBPs and it would be of interest to know if these co-evolved with the CaBPs and arose at the same time with the appearance of vertebrate species. Several CaBPs and calneurons have been shown to interact with and regulate various voltage-gated Ca^2+^channels (VGCCs) [28-36]. Despite the absence of CaBPs and calneurons in invertebrates these organisms possess VGCCs related to the major VGCC families in vertebrates. Drosophila, and C. elegans for example have genes encoding VGCC that are orthologues of the mammalian VGCCs alpha subunits that are regulated by CaBPs. Another known target for mammalian CaBP1, the IP3 receptor [37-39] is also present in invertebrates. Caldendrin has been shown to interact with the protein Jacob [40] and interestingly, this protein is also a vertebrate-specific protein. The N-terminus that distinguishes caldendrin from the splice variants CaBP1S and CaBP1L is not present in species below mammals. Jacob can, however, bind the EF-hand core domain that is present in CaBP1 as well as caldendrin [40] and it is possible, therefore, that in lower vertebrates Jacob is regulated by CaBP1 or even other CaBPs.

## Discussion

This study has examined the presence of genes encoding CaBP/calneuron proteins in a range of species. Based on relative sequence identity, pattern of EF hand inactivation and phylogenetic analysis the calneurons appear to be a distinct subfamily from the CaBPs. We found that no CaBP/calneuron orthologues could be found in invertebrate species, in early deuterosomes or in lampreys and hagfish. The presence of at least 5 CaBP/calneurons was detected in the elephant shark genome database but the sequences were too fragmentary for further analysis. In the case of the teleost zebrafish the whole family of CaBP/calneurons was present. We have also examined evolutionary changes in CaBP/calneuron protein sequences in vertebrates. Calmodulin is highly conserved in all organisms and even where there are multiple calmodulin genes in a species these encode an identical sequence [41]. When vertebrates diverged from invertebrates the calmodulin sequence became invariant. In contrast to calmodulin, other Ca^2+ ^sensor proteins have more specialised functions. Certain families of Ca^2+ ^sensor proteins show evidence of progressive increases in complexity during evolution with an increase in the number of distinct genes. This is typified in animals by the NCS protein family [6] and in plants by the CBL proteins [11,12]. It should be noted however that while there is a general increase in NCS genes from one in fungi to 14 in mammals for one subfamily, the GCAPs, there was also a transient expansion in their number in zebrafish compared to mammals [42,43]. In contrast to these protein families, it is likely that the CaBPs/calneurons arose as a full complement of genes prior to the separation of cartilaginous fish and the main subsequent increase in complexity stems from an increase in splice isoforms.

CaBP4 and CaBP5 are expressed predominantly in retinal photoreceptors where they regulate different VGCCs [14,34,44] and have been shown to play non-redundant roles in phototransduction in mammals based on studies on knockout mice [34,44]. In addition, mutation in CaBP4 in humans leads to congenital visual disorders [45,46]. Both CaBP4 and CaBP5 are present in zebrafish but we did not find them in the available genome sequences of amphibia (Xenopus) or birds. It is possible, however, that this is due to the fact that these genomes are not yet complete and contain gaps. There were significant differences in the N-terminus of CaBP4 in zebrafish compared to human CaBP4 suggesting that there may be some species differences in the regulation of photoreceptor function.

The pattern of active and inactive EF hands in the CaBPs/calneurons was no different in zebrafish compared to human proteins. EF hands 3 and 4 are predicted to be functional in CaBP2, 4 and 5 and are of the specific Ca^2+^-binding type (E at position 12 of the EF hand) in both zebrafish and human proteins. In contrast, the predicted functional EF hand 1 in CaBP2, 4 and 5 have D at position 12 in zebrafish but not in the human CaBP2 and 4 proteins suggesting that there is a change from Mg^2+^-binding to specific Ca^2+^-binding for EF hand1 in certain human CaBPs. This may have some functional significance as it could result in different structural effects of Ca^2+ ^binding in CaBP2 and 4 in zebrafish versus human proteins. Human CaBP1 short is the only member of the family to be characterised structurally [24,25]. Occupancy of EF hand 1 by Mg^2+ ^has a global effect on the conformation of CaBP1 compared to the apo form of the protein and it was suggested to stabilise the protein in its interaction with target proteins in the absence of Ca^2+ ^[24] with more limited conformational changes on Ca^2+ ^binding to the other EF hands. Human CaBP2 and 4 would differ from CaBP1 as none of their functional EF hands are predicted to preferentially bind Mg^2+^. A further understanding of the consequences of the predicted differences in specificity of cation binding of the EF hands of CaBP proteins will require resolution of additional structures of human and zebrafish proteins.

One general issue about the CaBPs is the extent to which they have overlapping or non-redundant roles. In addition, despite their relatively low level of sequence identity with calmodulin they do bind to some common targets such as VGCCs although the physiological effects on the VGCCs differ [47]. The existence of the neuronally expressed CaBPs (1 and 2) and calneurons in all vertebrates suggests that they have functionally important and potentially non-redundant roles. Particularly noteworthy is the high level of conservation of calneuron 1 and CaBP1S which have 93.1 and 86.8% identity between zebrafish and human proteins. Again this argues for significant conserved functions. The only characterised target for calneurons is the enzyme phosphatidylinositol-4-kinase (PI4K) IIIβ. Both calneurons bind to and inhibit this enzyme which is required to generate inositol lipids needed for vesicular traffic from the Golgi complex. Orthologues of PI4KIIIβ have similar roles in yeast [48] and flies [49] and so this inhibitory control by the calneurons is a more recent regulatory mechanism. To date, however, there are no knock-outs of CaBP1, CaBP2 or the calneuron genes in any species that would allow a test of the non-redundancy of their functions.

## Conclusions

The analyses reported here are consistent with a designation of the calneurons as a distinct sub-family of the CaBPs. In the case of other families of EF-hand containing proteins there is evidence for a progressive increase in the family number during evolution. In contrast the gene number for CaBPs/calneurons does not increase from at least teleost fish (and perhaps from cartilaginous fish) onwards. A distinctive evolutionary change in these proteins in vertebrates has instead been an increase in the number of splice variants present in mammals.

## Abbreviations

CaBP: calcium-binding protein; CBL: calcineurin B-like protein; GCAP: guanylyl cyclase-activating protein; KChIP: Kv channel-interacting protein; NCS: neuronal calcium sensor; VGCC: voltage-gated calcium channel; VILIP: visinin-like protein.

## Competing interests

The authors declare that they have no competing interests.

## Authors' contributions

HVM carried out bioinformatic analysis and interpretation of the data and contributed to the preparation of the manuscript. LPH contributed to the interpretation of the data and the writing of the manuscript. RDB carried out bioinformatic analysis, interpretation of the data and prepared the final version of the manuscript. All authors read and approved the final version of the manuscript
